# Thrombotic microangiopathy after traumatic brain injury: A case report and review of the literature

**DOI:** 10.1002/ccr3.7838

**Published:** 2023-09-08

**Authors:** Xavier Van Meerbeeck, Leen Janssen, Rowena Vleut, Philip Verdonck, Alain Gadisseur, Rudi De Paep, Walter Verbrugghe, Philippe Jorens

**Affiliations:** ^1^ Department of Intensive Care Medicine Antwerp University Hospital, University of Antwerp Edegem Belgium; ^2^ Department of Nephrology Antwerp University Hospital, University of Antwerp Edegem Belgium; ^3^ Department of Emergency Medicine Antwerp University Hospital, University of Antwerp Edegem Belgium; ^4^ Department of Hematology Antwerp University Hospital, University of Antwerp Edegem Belgium

**Keywords:** coagulation, DIC, plasmapheresis, thrombotic microangiopathy, trauma, neurotrauma

## Abstract

**Key Clinical Message:**

This case report supports that trauma can rarely cause thrombotic microangiopathy (TMA). Early recognition is important due to a high mortality of untreated TMA, but diagnosis can be delayed by attributing lab abnormalities as due to blood loss.

**Abstract:**

Major trauma can provoke coagulopathy, ranging from hypo‐ to hypercoagulation. Thrombotic microangiopathy (TMA), characterized by hemolytic anemia, renal failure, thrombocytopenia, and intravascular hemolysis, results in bleeding tendency but also microvascular thrombosis. We report a rare case of isolated traumatic brain injury leading to TMA treated with plasmapheresis.

## Graphical abstract

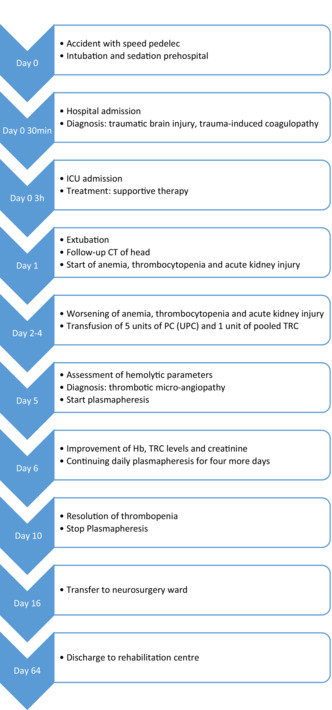

## 1 INTRODUCTION

Trauma‐induced coagulopathy (TIC) occurs in 25–35% of severe trauma patients and is associated with an increased incidence of bleeding, blood transfusion, multi‐organ failure, and even death.^1^ It is a complex and convoluted process, not completely understood, but believed to arise from endothelial and platelet dysfunction, hypofibrinogenemia or dysfibrinogenemia and hyperfibrinolysis, and is exacerbated by acidosis, hypothermia, hemodilution, and factor consumption related to blood loss and fluid resuscitation.^1^ Recently, TIC is now more appropriately renamed “coagulopathic response to trauma” because the coagulopathy is not a uniform phenotype, with a thrombotic phenotype at one end, a bleeding phenotype at the other, and even a mixed thrombotic‐bleeding phenotype along the spectrum. Therefore, it usually presents immediately after the injury with an hypocoagulable phenotype, marked by hypofibrinogenemia and hyperfibrinolysis. TIC, however, can also present as a hypercoagulable phenotype, for example, presenting with fibrinolysis shutdown, and can show dynamic changes over time, evolving from one phenotype to another over hours or days.^2^

Remarkably, the so‐called thrombotic microangiopathy (TMA) is another potentially fatal coagulation disturbance leading to systemic anticoagulation with higher bleeding tendency but at the same time microvascular thrombosis. It is caused by a variety of conditions and is characterized by thrombocytopenia, microangiopathic hemolytic anemia (MAHA), and signs of organ damage such as acute kidney injury (AKI), neurologic or gastrointestinal symptoms.^3^ Classically thrombotic thrombocytopenic purpura (TTP), atypical hemolytic uremia syndrome (HUS), and Shiga toxin E‐coli related (STEC) HUS are well‐known subtypes of this disorder. Recently, trauma has been suggested as a trigger for initiating TMA.^4^

Progression of TIC to TMA, however, has not been described and only a few case reports^5^, ^6^, ^7^ and one case series^8^ describe TMA following trauma. We report an additional patient with TMA following an isolated traumatic brain injury.

## 2 CASE REPORT

A previously healthy 61‐year‐old patient was hospitalized after he suffered a major head injury following an accidental fall with his speed pedelec. On arrival of the emergency medical services, he presented with an obstructed airway and his Glasgow Coma Scale (GCS) was 7 (Eye response 1, Verbal response 2, Motor response 4 (EMV)). A large scalp wound was seen, but no other external injuries were observed. The airway was secured prehospital after the administration of sedatives. One gram of tranexaminic acid was administered intravenously (IV), full spinal immobilization was applied and he was transported to our trauma center, all as per local protocol. During the primary survey, one unit of packed red blood cells of unmatched O negative was administered as tachycardia (heart rate 120 beats per minute (bpm) [60–100 bpm]) was seen with an actively bleeding scalp wound. Rotational thromboelastometry (ROTEM) showed a fibrin clot obtained by platelet inhibition with cytochalasin D amplitude at 10 min (FIBTEMA10) of 5 mm [6‐12 mm], an extrinsically activated test amplitude at 10 min (EXTEMA10) of 42 mm [43–63 mm] which suggest a decreased fibrinogen,^9^ for which 3 g of fibrinogen were administered IV. Blood analysis at hospital admission showed a hemoglobin (Hb) of 14.5 g/dL [normal value of 13.3–17.1 g/dL], platelets of 208 x 10^9^/L [166–396 x 10^9^/L], and a decreased fibrinogen level of 157 mg/dL [170–420 mg/dL], confirming the FIBTEM results. International normalized ratio (INR) and activated partial prothromboplastin time (APTT) were within normal limits: 1.10 [0.9–1.2] and 24.6 seconds (s) [23–31 s], respectively. His blood type was B positive.

Whole body computed tomography (CT) showed a complex skull and skull base fracture, bilateral intraparenchymatous frontal, temporal, and occipital hemorrhagic contusions, a left‐sided subdural hematoma, and bilateral diffuse subarachnoid hemorrhages. No intracranial aneurysm was detected on the CT‐angiography. No other injuries were found.

The patient was admitted to the intensive care unit for supportive TBI therapy. Amoxicillin‐clavulanate was started for prophylaxis of aspiration pneumonia. The patient was weaned and extubated on day 1 but his consciousness remained impaired with a GCS of 9 (E2M5V2) and he remained agitated for which he received a low dose of dexmedetomidine. A follow‐up CT showed a minimal increase of the hemorrhagic contusion zones but no other changes, in particular no major swelling of the brain. In the next few days, the patient developed anemia (Hb nadir 6.7 g/L) and thrombocytopenia (platelets nadir 31.10^9^/L) (Table 1). This thrombocytopenia was initially attributed to dilution and consumption due to the blood loss observed during the primary survey, but over the next few days, no ongoing macroscopic blood loss could be observed. A total of 5 cross‐matched B‐positive units of packed red blood cells were transfused over a three‐day timespan between day 2 and day 4 of the ICU stay and 2 units of pooled O‐positive platelets on day 2 and 3, respectively, without an observed increase in hemoglobin or platelet count. To rule out active bleeding or mechanical destruction by shear stress due to injury to the large vessels we performed a CT‐angiography of the neck, thorax, and abdomen, which showed no active bleeding site nor any damage to the large vessels.

**TABLE 1:** Overview of all relevant hematological and biochemical parameters in the peripheral blood.

	Normal range	Day 0	Day 1	Day 2	Day 3	Day 4	Day 5^a^	Day 6	Day 7	Day 13	Day 20	Ambulatory
**Hematology**												
Hemoglobin (g/dL)	13.3–17.1	14.5	12.9	8.4	7.2	7.3	7.0	6.7	7.9	7.2	9.1	14.5
Platelets (10^9^/µL)	166–396	208	140	32	35	52	31	36	83	262	351	206
Schistocytes (%)	<1						6	7	5	3	3	<1
Plasma hemoglobin mg/L)	10–40						559					
Haptoglobin (g/L)	0.40–2.80					0.04		0.11	0.52	2.65		1.57
**Hemostasis**												
APTT (s)	23–31	24.6	22.9	28.2	26.3	23.6	22.2	22.8	<20.0	25.6	20.3	23.4
PT (%)	78–123	81	85	100	117	121	108	97	85	111	106	120
INR	0.9–1.2	1.10	1.08	1.02	0.95	0.94	0.98	1.03	1.08	0.96	0.98	0.93
Fibrinogen (mg/dL)	170–420	157	182	300	473	527	589	300	207	585		295
ADAMTS‐13 activity (%)	61–131						32					67
**Hemostasis**												
Serum creatinine(mg/dL)	0.60–1.10	1.01	1.1	2.65	3.84	5.51	6.89	6.40	6.01	2.18	0.91	0.78
e‐GFR CKD‐EPI (mL/min/1.73 m^2^)	>60	80	72	25	16	10	8	9	9	32	91	97
CRP (mg/L)	<10	<4	69	202	222	190	179	55.6	15.9	184	38	
LDH (E/L)	120–246	434	466	1371	2612	3195	3421	831	398	301	227	171
CK (E/L)	46–171	277	284	502	937	991	1258	305	80	50	34	37
Total bilirubin (mg/dL)	0.30–1.20	0.51	1.3	3.8	4.8	4.0	4.7	2.7	1.9	0.65	0.22	0.29
Unconjugated bilirubin (mg/dL)	0.10–1.20	0.38	0.91	3.4	4.3	2.9	3	1.7	1.3	0.36	0.11	0.29
**ANF**	Negative						Negative					
Anticardiolipin												
IgG (U/mL)	<10						0.7					
IgM (U/mL)	<10						2.4					
Serum electrophoresis	Normal pattern						Abnormal peak in β2 fraction					Normal pattern
Albumin (g/L)	31.8–51.2						26.2					42.4
Alpha‐1 (g/L)	1.7–4.0						5.8					2.9
Alpha‐2 (g/L)	4.0–9.7						5.1					7.7
Beta‐1 (g/L)	2.7–5.9						2.3					3.9
Beta‐2 (g/L)	1.8–5.3						6.0					3.2
Gamma (g/L)	6.3–15.4						4.6					9.0
Immunofixation	Negative						Negative					
**Complement**												
Total hemolytic complement (%)	69–129%						120					
C3 (g/L)	0.9–1.7						1.16					
C3d (g/L)	<0.0075						0.0074					
C4 (g/L)	0.12–0.36						0.26					
CMV												
IgG (E/mL)	<0.5						2.7					
IgM	Negative						Negative					
HIV serology	Negative						Negative					
Vitamine B12 (ng/L)	211–911						484					

Abbreviations: ADAMTS13, a Disintegrin and Metalloproteinase with Thrombospondin motifs 13; ANF, antinucleoid factor; APTT, activated partial prothromboplastin time; BUN, blood urea nitrogen; CK, Creatinine kinase; CMV, cytomegalovirus; Cr, creatinine; CRP, C reactive protein; e‐GFR, estimated glomerular filtration rate; HIV, human immunodeficiency virus; INR, international normalized ratio; LDH, lactate dehydrogenase; PT, prothrombin time.

^a^ Plasmapheresis was started on day 5.

We also observed an acute kidney injury with a creatinine of maximal 6.89 mg/dL [0.6–1.1 mg/dL] on day 5 with sustained diuresis, an increased lactate dehydrogenase (LDH), and unconjugated bilirubin (Table 1) and red‐brown urine. Additional hemolytic parameters were therefore determined and were significant for a decreased haptoglobin of 0.04 g/L [0.40–2.8 g/L], a positive direct Coombs test and the presence of 6% schistocytes [normal <1%) in the peripheral blood smear. C‐reactive protein (CRP) level was also elevated (maximally 222 mg/L [<5 mg/L], but no fever was observed. (Table 1).

Because of the presence of microangiopathic anemia, thrombocytopenia, and signs of end‐organ failure (renal failure), the diagnosis of TMA was made.^3^, ^10^, ^11^ Additional blood work to screen for etiologies was obtained prior to treatment.

After premedication with methylprednisolone, 100 mg IV plasmapheresis of 1.5 times the total plasma volume was started on day 5 of hospitalization. In the following days, we observed an increase in platelets and a dramatic decrease in LDH, creatinine, bilirubin, and CRP (Table 1). Plasmapheresis was continued for five consecutive days. At this point Hb was 7.4 g/dL, platelets were 202 × 10^9^/L, creatinine was 3.86 mg/dL and haptoglobin was normalized. The patient was further monitored but there was no relapse of molytic anemia, thrombocytopenia, or acute kidney injury. There was a transient rise of CRP on day 12, which could be attributed to nosocomial pneumonia which was treated successfully with piperacillin‐tazobactam.

On day 5 after the trauma, findings of an additional extensive blood work included a reduced a disintegrin and metalloproteinase with a thrombospondin type 1 motif, member 13 (ADAMTS13) (32% [normal 61–131%]) which further supported our diagnosis; a negative antinuclear factor (ANF) and anticardiolipin antibodies; an abnormal peak in beta‐2 fraction on protein electrophoresis (which can be explained by the administration of IV iodium contrast prior that day); a negative immunofixation; normal complement factors; a normal concentration of Vitamin B12; and negative serology for cytomegalovirus and human immunodeficiency virus (Table 1).

**FIGURE 1:**
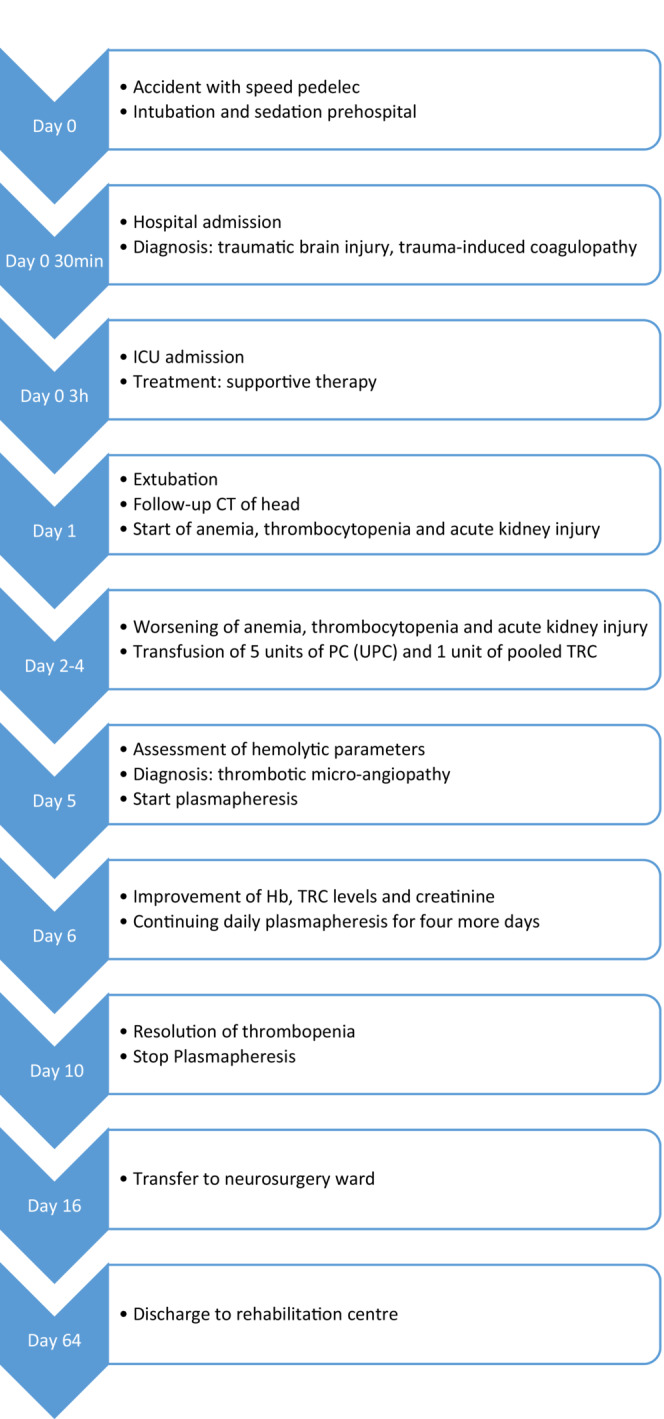
Timeline of events.

The patient could be transferred to the neurosurgery ward on day 16 for further care and active multidisciplinary rehabilitation. His neurologic status improved to a GCS of 14 (E4M6V4) but he suffered persisting impaired vision in both eyes, due to the location of the trauma. On day 66 after the admission he was discharged to a rehabilitation center. Blood analysis on discharge did not show any remarkable abnormalities, in particular a recovered kidney function to baseline, an absence of thrombocytopenia, and mild anemia (Hb 12.5 g/L) (Table 1). A blood work obtained ambulatory after discharge, showed a normal ADAMTS13 activity level of 67% (which underlies the fact that the decreased activity had been temporary) and an absence of anemia, thrombopenia, or kidney failure (Table 1). A timeline of the above‐mentioned events during hospitalization is shown in Figure 1.

## 3 DISCUSSION

TMA is a hematologic condition, characterized by the formation of microthrombi in small vessels due to endothelial cell damage and the subsequent mechanical intravascular fragmentation of red blood cells by flow through fibrin and platelet deposits. Laboratory features consist of anemia, thrombocytopenia, increased creatinine, and the presence of fragmented red blood cells (schistocytes) on peripheral blood smear.^3^

The classic presentation consists of thrombocytopenia, Coombs negative (mechanical) hemolysis, and signs of end‐organ damage. Early diagnosis is imperative as the disease carries a high mortality and urgent treatment is mandatory.^12^ In our patient, the diagnosis was delayed because thrombocytopenia and anemia are common in the posttraumatic setting and can also be attributed to blood loss, volume expansion, consumption thrombocytopenia and trauma‐induced disseminated intravascular coagulation (DIC). The acute kidney injury, however, could not be explained by the trauma as there was no renal injury observed on CT and the kidney function did not respond to intravenous fluid administration. There were no gastro‐intestinal symptoms and the altered mental status could be attributed to the brain injury too.

The etiology of TMA is broad and can include: infections (STEC in HUS; Streptococcal pneumonia infection, HIV, parvovirus, CMV); Thrombotic thrombocytopenic purpura (TTP), caused by a severe ADAMTS13‐deficiency; abnormal activation of the complement system in atypical HUS, malignancy, solid organ transplantation; exposure to certain drugs; pregnancy; Vitamin‐B12 or folate deficiency; auto‐immune diseases; antiphospholipid syndrome; or malignant hypertension.^3^, ^13^, ^14^, ^15^, ^16^ These major causes of TMA, however, are unlikely as precipitating TMA in our case as described further. Trauma, however, is not included in the classic etiology in many reviews and has yet only recently been recognized as a potential mechanism of secondary TMA.^4^

We could only identify three case reports handling TMA following major trauma^5^, ^6^, ^7^ The first case described TMA following blunt abdominal liver injury in a 23 year‐old male who suffered a motor vehicle accident. The diagnosis was only made after 21 days and following treatment for sepsis and start of hemodialysis,^6^ which is longer and more complicated than in our case. Moreover, drug‐induced TMA could not be ruled out, as a full list of administered medication was not disclosed.^5^ Ikegami et al. described TMA in an 81‐year‐old woman following a traumatic pelvic fracture and who received a transcatheter arterial embolization. After 5 days of hospitalization, a diagnosis of TMA was made on the basis of low thrombocyte count, presence of schistocytes (28.8%), AKI requiring dialysis, fever, delirium, and discarding alternative diagnoses. The time frame of the case is consistent with our case, but an important difference is that our patient had a sustained diuresis during the hospitalization.^5^ More recently, Riley et al. described microangiopathic hemolytic anemia in a patient following a motor vehicle accident, but was in further work‐up diagnosed with scleroderma based on findings on the kidney biopsy, which can also be a cause of TMA.^7^

Further, “probable” TMA following trauma (t‐TMA) was described in a retrospective single‐center case series of patients admitted to the ICU between 2018 and 2019. Of 1164 screened patients 20 (1.7%) were categorized as having t‐TMA by meeting several diagnostic criteria: having a recent trauma, having transfusion refractory thrombocytopenia, laboratory abnormalities suggestive of TMA, and finally exclusion of other causes of TMA. 17 of them received total plasma exchange.^8^ In the case series three patients were described as having an abbreviated injury score (AIS) of ≥3 in the head, but the exact location of the trauma or the concurrence of other regions like chest or abdomen is not described. Therefore, to our knowledge, this is the first description of TMA following isolated traumatic brain injury.

Because of the rarity of the disorder we initially considered alternative diagnoses. Mechanical destruction by damage to the large vessels due to the trauma was ruled out by a CT‐angiography. The normal to elevated fibrinogen level from day 1 and onwards, the normal prothrombin time and activated partial thromboplastin ruled out DIC. The direct Coombs was positive but the interpretation is challenging in the setting of trauma, as urgent transfusions of O‐negative units in non‐O patients can result in a false positive test, which happened in our patient. Transfusion‐mediated hemolytic anemia can therefore not be fully excluded. Drug‐induced TMA is also a possibility given the numerous drugs that were administered, but seems unlikely because none of the administered drugs (Table 2) are known to have an association with the occurrence of TMA.^15^, ^17^ Although the CRP level was high at diagnosis, infection seems unlikely because of the absence of fever or hemodynamic instability, negative viral serology and dramatic decrease of CRP after starting plasmapheresis without escalation of the antibiotic therapy. Level of ADAMTS13 was reduced to 32%, which is insufficient for the diagnosis of primary TTP. Complement factors were within normal ranges. There was no history of malignancy or solid organ transplantation. Protein electrophoresis and immunofixation did not show clues for an underlying hematologic malignancy. Auto‐immune serology was negative. Antiphospholipid markers were negative ambulatory.

**TABLE 2:** List of administered medication up to diagnosis of thrombotic microangiopathy.

Medication	Days administered
Rocuronium	0
Ketamine	0
Midazolam	0
Fibrinogen	0
Tranexaminic acid	0
Propofol	0–1
Remifentanil	0–1
Noradrenaline	0–1
Fentanyl	0–5
Paracetamol	0–5
Haloperidol	1–4
Dexmedetomidine	1–5
Tramadol	2–5
Furosemide	4–5
Morphine	5
Clonidine	5

The pathophysiology of TMA in the setting of trauma is not well understood but a relationship with TIC seems likely based on shared pathophysiologic mechanisms. In trauma, it has been suggested that an overload and prolonged circulation of ultra‐large vWF multimers (ULvWF), caused by endothelial damage, exceeds the protein cleaving capacity of ADAMTS13.^18^, ^19^, ^20^ Particularly in brain injury vWF is hypothesized to have an impact on the integrity of the blood–brain barrier and ADAMTS13 playing a protective role in the endothelial integrity.^21^ The subsequent reduction in ADAMTS13 activity in trauma has been associated with an increased need for transfusion, increased incidence of coagulopathy, organ failure and mortality^18^, ^20^, ^22^, ^23^ Disturbances of the vWF/ADAMTS13 ratio have also been suggested in the pathophysiology of micro‐angiopathic kidney injury following trauma^24^ and restoring this disturbance can lead to a reduction of organ failure.^22^ The reduced ADAMTS13 activity in our patient is compatible with the described pathophysiological mechanisms and the low fibrinogen at admission supports a TIC. We hypothesize that due to the brain injury, TIC developed as supported by the decreased level of fibrinogen at admission, but evolved into TMA by disturbance of the vWF/ADAMTS13 ratio by overproduction of ULvWF caused by endothelial cell damage, platelet activation, and disruption of the blood–brain barrier.

Untreated TMA has a high mortality, even up to 90%. Treatment consists of urgent plasma exchange by removing excessively thrombogenic and anti‐fibrinolytic molecules and to replenish the deficient anticoagulants and profibrinolytic molecules in order to regain a normal homeostatic milieu.^25^ In this patient a rapid normalization of the thrombocytopenia, acute kidney injury, and hemolysis was observed after starting of PE, which further supports the diagnosis of a TTP‐like TMA.

To our knowledge, this is only the fourth case report of TMA secondary to trauma and the first to do so following TBI. The role of trauma in the pathophysiology of TMA has only recently been appreciated. A relationship with TIC seems likely based on shared pathophysiologic mechanisms but is not extensively described. The condition is extremely rare, but the prevalence may be underestimated by the attribution of thrombocytopenia and anemia to blood loss, consumption, or DIC. Trauma‐induced TMA should however be considered in the case of transfusion refractory thrombocytopenia and signs of hemolysis. Early recognition is important as the untreated mortality of TMA is high and total plasma exchange should therefore not be delayed.

## AUTHOR CONTRIBUTIONS

**Xavier Van Meerbeeck:** Investigation; methodology; writing – original draft; writing – review and editing. **Leen Janssen:** Conceptualization; data curation; project administration; writing – original draft; writing – review and editing. **Rowena Vleut:** Formal analysis; methodology; writing – review and editing. **Philip Verdonck:** Formal analysis; validation; writing – original draft; writing – review and editing. **Alain Gadisseur:** Investigation; supervision; writing – review and editing. **Rudi De Paep:** Investigation; supervision; writing – review and editing. **Walter Verbrugghe:** Writing – review and editing. **Philippe Jorens:** Project administration; resources; supervision; writing – review and editing.

## FUNDING INFORMATION

The authors received no current or recent funding that might influence the work.

## CONFLICT OF INTEREST STATEMENT

The authors declare that there is no conflict of interest regarding the publication of this article.

## ETHICS STATEMENT

Written informed consent from the patient was obtained prior to submission of the case report and is available by the authors upon request.

## CONSENT

Written informed consent was obtained from the patient to publish this report in accordance with the journal's patient consent policy.

## Data Availability

The data that support the findings of this study are available on request from the corresponding author. The data are not publicly available due to privacy or ethical restrictions.
